# Comparative and Phylogenetic Analysis of Complete Chloroplast Genomes in *Leymus* (Triticodae, Poaceae)

**DOI:** 10.3390/genes13081425

**Published:** 2022-08-10

**Authors:** Zinian Wu, Chunyu Tian, Yanting Yang, Yuanheng Li, Qian Liu, Zhiyong Li, Ke Jin

**Affiliations:** 1 Institute of Grassland Research, Chinese Academy of Agricultural Sciences, Hohhot 010010, China; 2 Key Laboratory of Grassland Resources and Utilization of Ministry of Agriculture, Hohhot 010010, China

**Keywords:** *Leymus*, chloroplast genome, SSR, comparative analysis, phylogenetic relationship

## Abstract

*Leymus* is a perennial genus that belongs to the tribe Triticeae (Poaceae) which has an adaptive capacity to ecological conditions and strong resistance to cold, drought, and salinity. Most *Leymus* species are fine herbs that can be used for agriculture, conservation, and landscaping. Due to confusion taxonomy within genera, the complete chloroplast (cp) genome of 13 *Leymus* species was sequenced, assembled, and compared with those of three other previously published *Leymus* species (*Leymus condensatus*, *Leymus angustus*, and *Leymus mollis*) to clarify the issue. Overall, the whole cp genome size ranged between 135,057 (*L. condensatus*) and 136,906 bp (*Leymus coreanus*) and showed a typical quadripartite structure. All studied species had 129 genes, including 83 protein-coding genes, 38 transfer RNAs, and 8 ribosomal RNAs. In total, 800 tandem repeats and 707 SSR loci were detected, most of which were distributed in the large single-copy region, followed by the inverted repeat (IR) and small single-copy regions. The sequence identity of all sequences was highly similar, especially concerning the protein-coding and IR regions; in particular, the protein-coding regions were significantly similar to those in the IR regions, regardless of small sequence differences in the whole cp genome. Moreover, the coding regions were more conserved than the non-coding regions. Comparisons of the IR boundaries showed that IR contraction and expansion events were reflected in different locations of *rpl22*, *rps19*, *ndhH*, and *psbA* genes. The close phylogenetic relationship of *Leymus* and *Psathyrostachys* indicated that *Psathyrostachys* possibly is the donor of the Ns genome sequence identified in *Leymus*. Altogether, the complete cp genome sequence of *Leymus* will lay a solid foundation for future population genetics and phylogeography studies, as well as for the analysis of the evolution of economically valuable plants.

## 1. Introduction

*Leymus* Hochst is a significant perennial grass species of Triticeae (Poaceae) that is mainly distributed in Eurasia and North America [1,2] with strong adaptable characteristics to several environmental stressors such as drought, cold, and salinity [3]. Several species have been used for the genetic improvement of Triticeae cereal crops because of their larger spikes, higher grain yields, and better resistance to diseases and insects [4,5]. Nevertheless, its precise taxonomic status and the relationship among *Leymus* species are still debatable. Although morphological identification is relatively certain during the flowering period, it is difficult to perform morphological identification for roots, stems, and leaves in other growth stages, especially in the seedling stages. Initially, Nevski hypothesized that *Leymus* consisted of sect. *Leymus* and sect. *Anisopyrum* [6], but later on Tzvelev divided *Leymus* into four parts: sect. *Leymus* Hochst., sect. *Anisopyrum* (Griseb.) Tzvelev, sect. *Aphanoneuron* (Nevski) Tzvelev, and sect. *Malacurus* (Nevski) Tzvelev [7]. It has been confirmed that single-copy genes, the mitochondrial *coxII* intron, molecular markers containing AFLP, RAPD, and ISSR, as well as chloroplast intergenic spacers, are useful tools for *Leymus* species identification and these previous studies provide important evidence for the subdivision [8,9,10,11,12,13,14,15,16]; however, its complex evolutionary history remains unclear.

Chloroplasts (cp) are important semi-autonomous genetic organelles for the process of photosynthesis and carbon fixation [17,18]. The complete cp genome of most angiosperms has typically conserved quadripartite structures with a large single-copy (LSC) region, a small single-copy (SSC) region, and two copies of inverted repeat (IR) regions [18,19,20]. Few plants, such as some Leguminosae species, have cp genomes that are not the typical quadripartite structure owing to the loss of a reverse repetitive sequence [21]. CpDNA is predominantly inherited from the maternal parent; however, exceptions can be observed even though it rarely happens. The highly conserved cp genome can provide more reliable data for phylogenetic studies and is the optimal material for phylogeographic, system taxonomic, phylogenetic, and molecular evolution investigations [22]. To date, complete cp genomes are available for more than 100 Triticodae species, including five *Leymus* species. According to the evolutionary patterns of the cp genomes among 131 Triticodae species, *L. arenarius* (MK775256), *L. chinensis* (MK775258), and *L. triticoides* (NC_058745), *Psathyrostachys* species are believed to be closely related to the Eurasia *Leymus* species [23]. Although the cp genome is very significant, the number of published whole cp genome sequences of the *Leymus* species is still limited. Furthermore, some sequenced *Leymus* cp genomes have not been comprehensively and systemically studied.

The cp genome is uniparental, with large numbers of highly conserved and variable regions. Therefore, cpDNA sequences are valuable tools for determining plant barcoding and evolutionary relationships among plant species. Several cpDNA molecular markers, such as *trnL-trnF*, *trnH-psbA*, *trnK-rps16*, and *ndhF*, have been used to analyze the relationship among *Leymus* species and other Triticodae [8,11,13,14,24], and shed light into *Leymus* evolution. However, no systematic studies have been conducted on the development of cpDNA molecular markers of *Leymus*.

In this study, the cp genomes of 13 *Leymus* species were sequenced and analyzed along with publicly available data from three other specimens (*L. condensatus*, *L. angustus*, and *L. mollis*). Overall, the collected data were used to analyze and compare the genome characteristics, such as repeat sequences and IR boundaries. Moreover, cpDNA molecular markers for identifying *Leymus* germplasms were determined and the complete cp genomes were used to construct phylogenetic trees. Taken together, our results are expected to provide valuable genetic information, including new genetic markers for DNA barcoding, as well as enhance knowledge of the evolutionary relationships among *Leymus* species.

## 2. Materials and Methods

### 2.1. Sampling, DNA Extraction, and Genome Sequencing

The cp genomes of *Leymus arenarius* (MK775256.1), *Leymus duthiei* (NC_058748), and *Leymus komarovii* (NC_058744) were obtained from GenBank (Table S1). Plant seeds whose accession numbers start with PI and W6 were obtained from the U.S. National Plant Germplasm System, and those that start with CF were collected by us. The cp genomes of 13 *Leymus* species were sequenced. Voucher specimens were deposited in the National Medium Term Genebank Forage Germplasm (Hohhot, China) (Supplementary Table S1). Total genomic DNA was isolated from fresh leaves using the CTAB method [25] and sequenced using an Illumina MiSeq platform with PE150 at Sangon Biotech (Shanghai, China).

### 2.2. Genome Assembly and Genome Annotation

Assembly of the cp genome sequences was performed using GetOrganelle and annotated using PGA [26,27] with manual corrections by Geneious v9.0.2 [28]. The circular cp genome map was visualized using Organellar GenomeDRAW [29]. The 13 assembled complete cp genomes were deposited in GenBank (Table S1).

### 2.3. Repeats and SSR Identification

Tandem repeats of *Leymus* cp genomes were identified using REPuter software, with hamming distance and minimal repeats set at 3 and 30, respectively [30]. MISA script was used to detect SSRs with the following parameter settings: 1–10 2–6 3–4 4–3 5–3 6–3 [31].

### 2.4. Comparative Genome Analysis

All 13 *Leymus* cp genomes were aligned using MAFFT v7.313 [32] with default settings. The BRIG [33] and mVISTA [34] software were used to compare the variations among all available *Leymus* cp genomes, with *L. chinensis* being set as the reference sequence. In addition, IRScope was used to compare and visualize the junctions and borders of the IR regions [35]. The nucleotide diversity (Pi) rates of sequence divergence between *Leymus* species were calculated using DnaSP v6.12 [36].

### 2.5. Phylogenetic Analysis

The complete cp genomes and shared protein-coding genes of the 13 newly sequenced *Leymus* species were used for phylogenetic analysis, along with those published in NCBI, which included those of three previously sequenced *Leymus* species (Table S1) and the outgroups *Brachypodium distachyon* (NC_0110320) and *Oryza sativa* (NC_011032). All datasets were aligned using MAFFT v7.313 [32] with default settings. ModelFinder was employed to find the best model in PhyloSuite v1.2.2 [37]. The best substitution model based on the complete cp genomes was TVM + F + R2 for the maximum likelihood (ML) and GTR + F + I + G4 for the Bayesian inference (BI) analyses, and the best substitution model based on the shared protein-coding genes was GTR + F + R3 for the maximum likelihood (ML) and GTR + F + I + G4 for the Bayesian inference (BI) analyses, which were performed using RAxML v8.2.11 [38] with 1000 non-parametric bootstrap replicates and MrBayes v3.2.6 [39], respectively.

## 3. Results

### 3.1. Characteristics of the Leymus Species Complete Chloroplast Genomes

**Figure 1:**
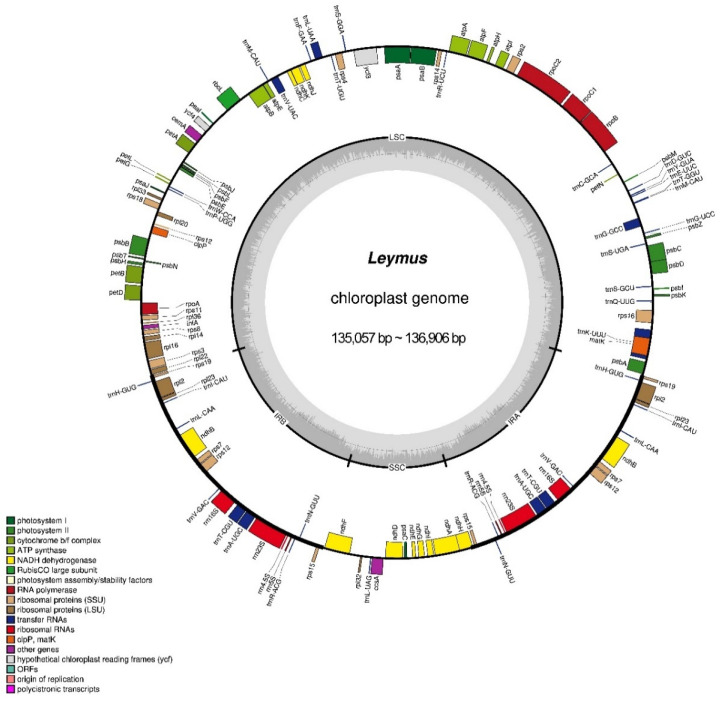
Chloroplast (cp) genome map of *Leymus* species with *L. chinensis* as reference. The inner circle shows the GC and AT content. LSC, SSC, and IR (IRa and IRb) represent the large single copy, small single copy, and inverted repeats, respectively. Genes inside of the large circle are transcribed clockwise and those outside are transcribed counterclockwise. Genes with different functions are shown in different color blocks.

The cp genomes of the *Leymus* species had the typical quadripartite structure, with the length of the LSC, SSC, and IR regions ranging between 80,149 bp in *L. triticoides* and 81,053 bp in *L. paboanus*, 12,708 bp in *L. karelinii* and 12,797 bp in *L. duthiei*, and 20,813 bp in *L. angustus* and 21,578 bp in *L. komarovii*, respectively (Table S1). The size of the 16 *Leymus* cp genomes ranged from 135,057 bp of *L. condensatus* and 136,906 bp of *L. coreanus* (Figure 1, Table S1) and comprised 129 genes, including 83 protein-coding genes, 38 transfer RNA (tRNA) genes, and 8 ribosomal RNA (rRNA) genes (Table 1 and Table S2). Among these genes, six protein-coding genes, four rRNAs, and nine tRNA were the same in the two identified IR regions; in other words, these 19 genes were duplicated in the two IR regions. The LSC region contained 62 protein-coding genes and 20 tRNA genes, whereas 11 protein-coding genes and one tRNA gene were located in the SSC regions. Among all genes, seven protein-coding genes and six tRNAs were within one intron; additionally, two protein-coding genes (*rps12* and *ycf3*) were within two introns (Tables S2 and S3). In particular, *rps12* was trans-spliced, with the 5′-end–exon being located in the LSC region, and two 3′-end–exons being both in the IRs. The GC content of the cp genomes ranged from 38.33–38.42%, which was almost identical to that of all other *Leymus* species (Table S2), with the IR regions having the highest GC content (43.89–44.01%), followed by the LSC (36.35–36.41%) and SSC regions (32.23–32.63%). Noteworthily, the two IR regions had the same GC content (Table S2).

### 3.2. Analysis of Repeat Sequences and SSRs

**Figure 2:**
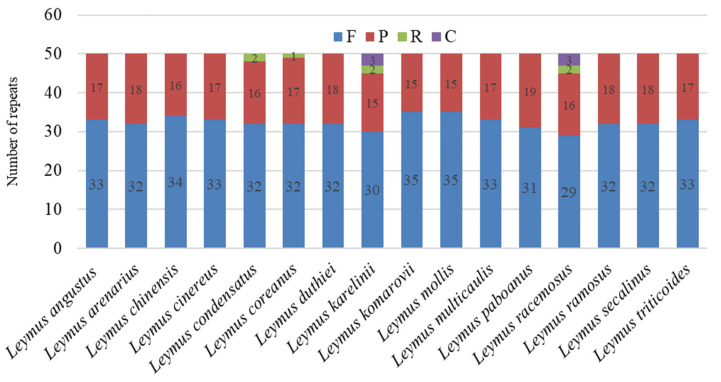
The number of the four types of repeats identified in the 16 Leymus cp genomes. F, P, R, and C represent forward, palindromic, reverse, and complementary repeats, respectively.

All 16 *Leymus* cp genomes comprised 800 repeats, which consisted of 518 forward (F), 269 palindromic (P), seven reverse (R), and six complementary (C) repeats (Figure 2). The total number of repeats was consistent for each *Leymus* species, whereas the repeat type and distribution were different. *L. condensatus* and *L. coreanus* contained three repeat types, and *L. karelinii* and *L. racemosus* contained four repeat types, whereas all other species contained two repeat types. Among them, F repeats accounted for the biggest proportion (58–70%), followed by P repeats (30–38%), R repeats (*L. condensatus*, *L. coreanus*, *L. karelinii*, and *L. racemosus* had 4%, 2%, 4%, and 4%, respectively), and C repeats (6% in *L. karelinii* and *L. racemosus*) (Figure 2). The length of the repeats in all 16 *Leymus* cp genomes analyzed ranged from 30 to 286 bp, with most repeats being between 34 and 39 bp. The longest repeats were found in *L. duthiei* and *L. komarovii*.

**Figure 3:**
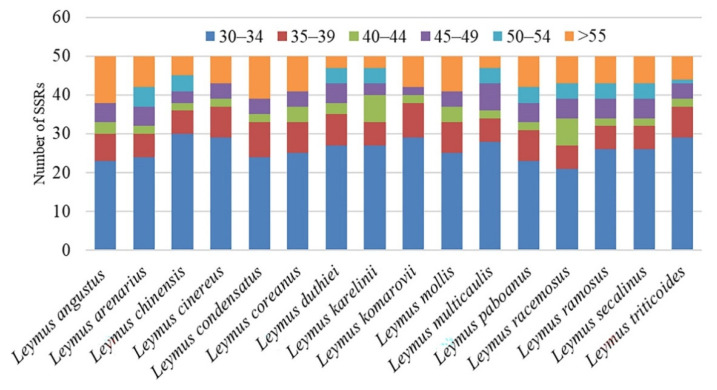
The number of repeats with different lengths in the 16 *Leymus* cp genomes.

In total, 707 SSRs were identified in the 16 *Leymus* cp genomes (Figure 3, Table 1). Mononucleotide repeats were the most abundant SSR among all *Leymus* species, accounting for 48.72–79.49% of all loci, followed by dinucleotide repeats (7.69–16.67%), trinucleotide repeats (4.88–7.69%), and tetranucleotide repeats (14.63–25.00%) (Table S4). Three pentanucleotide repeats were identified in *L. cinereus* and *L. triticoides*, two in *L. coreanus*, and one in *L. angustus*, *L. condensatus*, *L. komarovii*, and *L. mollis*. No hexanucleotide repeats were detected in the cp genomes. Most SSRs were composed of an A/T motif instead of a G/C motif. The pentanucleotide AAAAT/ATTTT and AAGAT/ATCTT repeats were identified in *L. cinereus* and *L. triticoides*, and the AATAT/ATATT repeat was found only in *L. coreanus* (Table S4).

The number of SSRs was not significantly different among the 16 *Leymus* cp genomes analyzed, ranging from 36 in *L. duthiei* to 50 in *L. coreanus* (Table 1). LSC was the region with more SSRs (mean 2.39 SSRs per kb), followed by the IR (mean 8.44 SSRs per kb) and SSC (mean 4.21 SSRs per kb) regions. Only two SSRs were located in the IRs (*rrna4.5*) of *L. angustus*, *L. condensatus*, and *L. mollis*, and the remaining species had 6–10 SSRs in the IR region (Figure 3, Table 1). Within the cp genomes of the 16 *Leymus* species, 56.76% and 67.35% SSR loci were found in the intergenic areas of *L. mollis* and *L. karelinii*, respectively. Moreover, the gene-coding regions of *L. karelinii* and *L. racemosus* had 20.41% and 27.03% SSR loci, and 11.11% and 16.67% SSR loci were found in the intron areas of *L. duthiei* and *L. chinensis.* (Table S5). What is needed to pay attention to is that SSR located in IGS (*trnR-UCU*, *rps14*), IGS (*atpB*, *rbcL*), IGS (*psbE*, *petL*), and IGS (*ccsA*, *ndhD*) only remain in *Leymus cinereus, Leymus arenarius, Leymus coreanus*, and *Leymus paboanus* (Supplementary Table S5)

### 3.3. Comparative Analysis of Genome Structure

**Figure 4:**
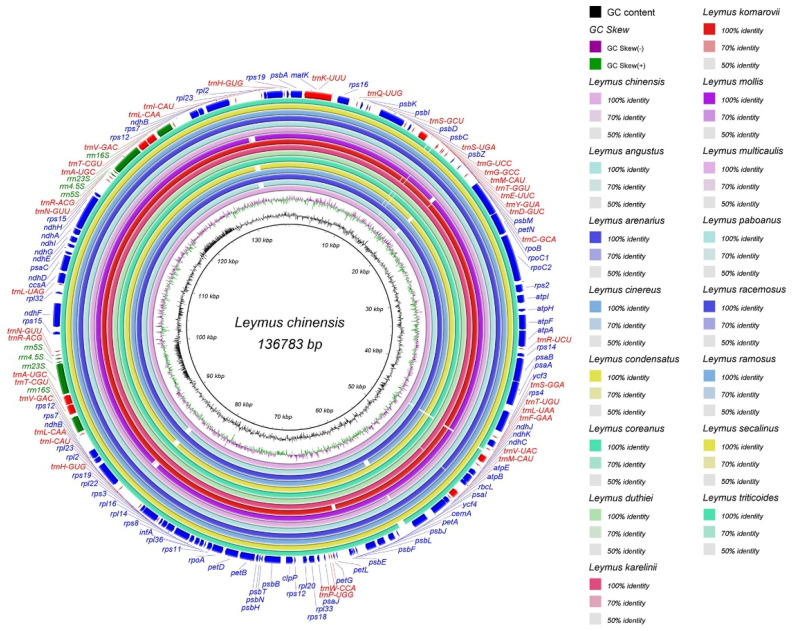
Sequence identity of the 16 *Leymus* species. The outermost circle represents the correspondence of protein-coding genes and intergenic spacer regions, and the innermost circle represents the *L. chinensis* genome as a reference. Other circles represent the output of the sequence identity analysis of the 15 *Leymus* species.

**Figure 5:**
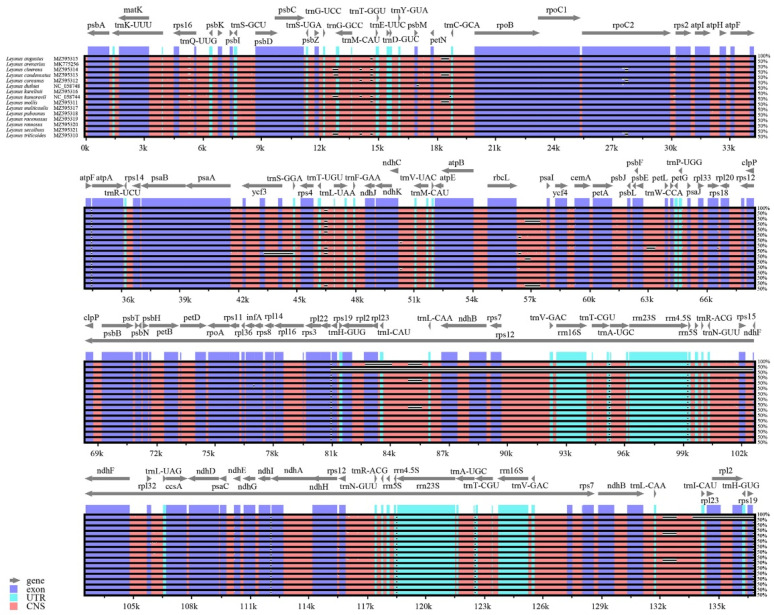
Global alignment of the cp genome sequences of the *Leymus* species using *L. chinensis* as reference. The value of the vertical scale (from 50% to 100%) represents the percentage of identity. Arrows indicate the annotated genes and their transcriptional direction. Different colored boxes represent exons, transfer RNA, ribosomal RNA, and non-coding sequences (CNSs).

**Figure 6:**
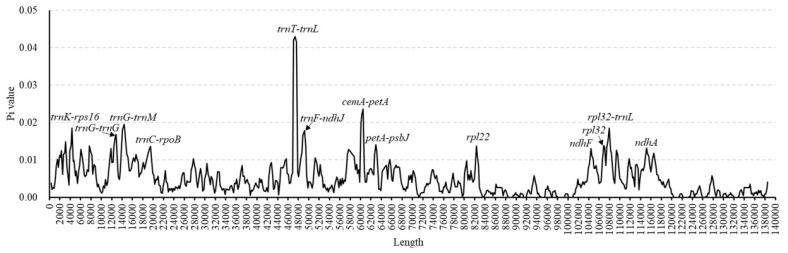
Nucleotide diversity (Pi) was determined by sliding window analysis of the aligned whole cp genomes of the 16 *Leymus* species (window length: 600 bp; step size: 200 bp). The *X*-axis represents the position of the midpoint of the window and the *Y*-axis represents the Pi of each window.

All aligned cp genome sequences were found to be highly similar, especially in the protein-coding and IR regions (Figure 4 and Figure 5). In this study, the average Pi values for the genome, coding DNA sequence (CDS), and intergenic spacers (IGS) among the 16 *Leymus* species were 0.0049, 0.0034, and 0.0081, respectively (Tables S6 and S7). Similar to other plants, coding regions were the most conserved among all *Leymus* species, and conservation of the IGS regions was secondary. In particular, the IR was the most conserved region, with merely a few hotspot regions, with the majority of the hotspot sections located in the LSC and SSC regions. In the 76 CDS regions, Pi% values ranged from 0 (*ndhE*, *petG*, *petN*, *psbF*, *psbI*, *psbN*, *psbT*, *psbZ*, *rpl36,* and *rps8*) to 0.0159 (*rpl32*), and the regions with large variations were *rps16*, *ndhH*, *matK*, *rpl22*, *psbM,* and *rpl32* (Pi% > 0.009) (Figure 6, Table S6). In the 121 IGS regions, Pi% values ranged from 0 (13 regions, Figure 6, Table S7) to 0.0431 (*trnT-trnL*), and the regions with large variations were *petG-trnW*, *rpl32-trnL*, *rpl22-rps19,* and *trnT-trnL* (Pi% > 0.03). These results indicate that these variable regions can be used as new genetic markers in DNA barcoding and phylogenetic studies of *Leymus* species.

### 3.4. Expansion and Contraction of the IR Region

**Figure 7:**
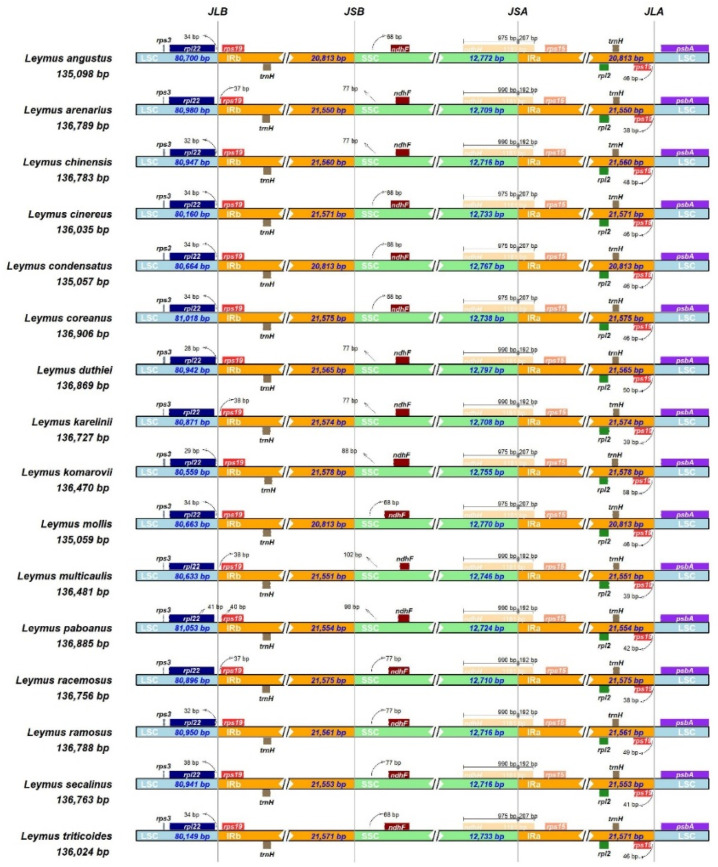
Comparison of the borders of the large single-copy (LSC), inverted repeat (IR), and small single-copy (SSC) junction boundaries in the cp genomes of the 16 *Leymus* species. JLB, JSB, JSA, and JLA represent the LSC/Irb, SSC/Irb, SSC/Ira, and LSC/Ira junction regions, respectively.

The expansion or contraction of the IR region differs among plant species; therefore, LSC, SSC, and IR boundary structures were analyzed among the 16 *Leymus* species. Only minor differences in the junction positions were detected. In all species, the junction of LSC/IRb (JLB) was located between *rp122* in LSC and *rps19* in Irb (Figure 7): *rp122* extended from 27 to 40 bp in length from LSC to Irb, and *rps19* extended from 37 to 57 bp in length from Irb to LSC. Similarly, the JLAs (junction of Ira/LSC) were located between *rps19* in Ira and *psbA* in LSC: *rps19* extended from 37 to 57 bp in length from Ira to LSC, and *psbA* extended from 81 to 91 bp in length from LSC to Ira. The *ndhF* gene deviated from the junction of the Irb/SSC (JSB) region, ranging from 68 to 108 bp in length. The *rps15* gene was located in the Irb, which ranged from 337 to 362 bp in length from the JSB border. Additionally, the *ndhH* gene of all *Leymus* species straddled the junction of the SSC/Ira (JSA) boundary regions, with 975 to 900 bp of the SSC region and 192 to 207 bp of the Ira region. Overall, the contraction and expansion events in Irs were reflected in the different locations of *rpl22*, *rps19*, *ndhH*, and *psbA* (Figure 7).

### 3.5. Phylogenetic Relationships between Leymus Species

**Figure 8:**
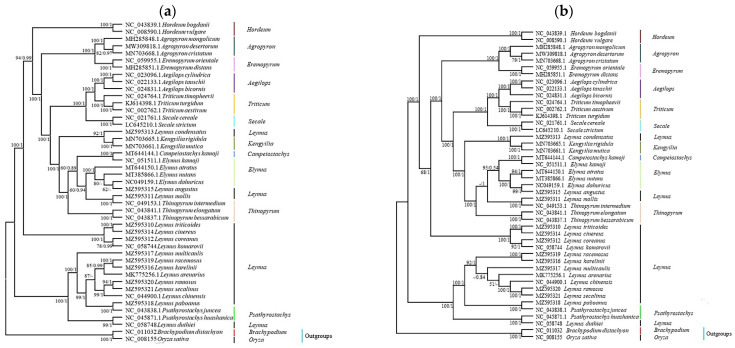
Phylogenetic tree of the *Leymus* species. *Brachypodium distachyon* and *Oryza sativa* Indica were selected as the outgroups. The tree was constructed by maximum likelihood and Bayesian inference with bootstrap values or posterior probabilities being indicated above the branches. The bootstrap values or posterior probabilities no more than 50% or 0.5 were shown in “-”. (**a**) Based on complete cp genome data; (**b**) based on 74 shared protein-coding genes.

Using *Oryza sativa* and *Brachypodium distachyon* as outgroups, cp complete genome and 74 shared protein-coding genes were used to perform phylogenetic analysis, respectively. The topological structure of the phylogenetic tree showed that 16 *Leymus* species were clustered in different clades with other 27 species across 11 genera of Triticum. In addition to *L. condensatus, L. angustus*, and *L. mollis,* the 13 *Leymus* species herein analyzed clustered along with two *Psathyrostachys* species in two clades. Two North American *Leymus* species (*L. triticoides* and *L. cinereus*) along with two Central Asia *Leymus* species (*L. coreanus* and *L. komarovii*) clustered into one clade and showed a close phylogenetic relationship. The other clade consisted of five Central Asia *Leymus* species (*L. racemosus*, *L. karelinii*, *L. multicaulis*, *L. ramosus*, and *L. paboanus*), one Eurasia species (*L. arenarius*), one East Asia species (*L. chinensis*), two Qinghai-Tibet Plateau *Leymus* species (*L. secalinus* and *L. duthiei*), and the two *Psathyrostachys* species (*P. juncea* and *P. huashanica*) (Figure 8).

## 4. Discussion

### 4.1. Genome Organization and Genome Features

*Leymus* is a genus of the botanical tribe Triticeae (Poaceae) comprising approximately 30 species and 19 subspecies, almost all of which are polyploid and cross-pollinating [1,2]. *Leymus* can be used for agriculture and conservation, and as an important genetic resource for Triticeae cereal crop improvement. In this study, 13 *Leymus* species were sequenced and comprehensively analyzed in comparison with three previously reported *Leymus* sequences. The cp genomes of all 16 *Leymus* species exhibited the typical quadripartite structure, which consisted of LSC, SSC, and two inverted repeats (IRa and IRb), and the cp genome size ranged from 135,057 bp (*L. condensatus*) to 136,906 bp (*L. coreanus*) (Figure 1, Table S2), which was in agreement with previously published data of Triticeae [23,40,41]. The cp genomes of *Leymus* species were highly conserved, with the same genomic structure and gene content. In particular, *accD*, *ycf1,* and *ycf2* genes coded in the *Leymus* cp genomes gradually degraded as compared to those of other plants, such as cattail (*Typha orientalis* Presl) and tobacco (*Nicotiana tabacum* L.) [42], and were similar to that of Triticeae species [23,43]. This event may be explained by the insertion and deletion of the no-triple base in the pairing sequence which may have occurred many times, causing a frameshift mutation and gene loss [42]. Moreover, since 20 genes with introns were identified in *Leymus* species, contrasting with *clpP* and *rpoC1* which had no introns, provides further evidence that the loss of *clpP* and *rpoC1* introns occurs in most Poaceae species [23,43,44].

SSRs, which are highly diverse in copy number, are important molecular markers that play a significant role in plant population genetics and evolution investigations [45,46]. A total of 707 SSR loci were identified in the 16 *Leymus* cp genomes (Figure 3, Table 1). Due to the rich A/T stretches of the repeats, the cp genome contained a high A/T content (Table S4). It is observed that the number of SSRs in the non-coding region was higher than that in the coding region (Table S5). These results are similar to those of other Triticeae species [23,47,48] and may be the reason why the mutation rate in IGS regions is higher than in the coding regions [49].

### 4.2. Comparative Analysis of Leymus

Compared with the coding region, the nucleotide diversity was higher in a non-coding region of the *Leymus* species. These results are consistent with other species [22,40,48,50]. Our study revealed 10 hypervariable regions in *Leymus* species: five coding regions (*rps16*, *ndhH*, *matK*, *rpl22*, *psbM*, and *rpl32*) and five non-coding regions (*petG-trnW*, *rpl32-trnL*, *rpl22-rps19,* and *trnT-trnL*) (Figure 6, Tables S6 and S7). Interestingly, the highly variable regions of *Leymus* species were different from those previously reported for designing phylogenetic trees and species identification of *Leymus*, such as *trnL-trnF*, *trnH-psbA*, *trnK-rps16*, *ndhF*, *rps16-trnQ*, *trnF-ndhJ*, and *ndhF-rpl32* [8,11,13,14,24,51,52,53]. Thus, by analyzing the variable regions of the 16 *Leymus* cp genomes, we were able to identify some molecular markers, such as SSRs [45,54] which can be more effectively used in DNA barcoding and to determine phylogenetic relationships in future studies.

### 4.3. Evolution and Origin of IRs

The IR regions of the cp genome are considered to be the most conserved regions [55]. Similar to previous studies on Triticeae, all 16 *Leymus* species had the same gene arrangement, in which six protein-coding genes (*rpl2*, *rpl23*, *rps12*, *ndhB*, *rps15,* and *rps19*) were duplicated [23,43,47]. Moreover, we identified the smallest reverse IR region in *L. angustus* (20,813 bp) and the largest in *L. komarovii* (21,578 bp) (Table S3). It was possibly the expansion and contraction of the IR regions, along with the spacer of a single copy, that caused variations in the length of the complete cp genomes [56]. Some reports have shown that slight expansion of the IR region may be caused by gene transfer, with double-strand break repair leading to wider expansion [57]. The different locations of *rpl22*, *rps19*, *ndhH,* and *psbA* may have been induced by IR contraction and expansion events in *Leymus* species, leading to changes in the IR boundaries among them. All *Leymus* species showed minor variations in junction positions of JLB (~13 bp), JSB (~34 bp), and JLA (~8 bp) (Figure 7). In addition, *ndhH* was found to straddle the JSA, which is consistent with previous data on other Triticeae species, such as *Agropyron*, *Hordeum*, and *Festuca* [23,47,58,59]. The different position of *ndhH* in the JSA border suggests that these variations may be caused by intramolecular recombination during early evolution [60,61,62].

### 4.4. Phylogenetic Relationships

*Leymus* has been widely recognized based on morphological, cytogenetic, and molecular data after Hochstetter separated it from the traditional *Elymus* L. in 1848; however, there is currently a lack of understanding of the evolutionary relationships among *Leymus* species. In this study, the whole cp genome sequences were used to perform phylogenetic analysis. Overall, the 16 *Leymus* species analyzed were clustered in different clades along with other 27 species across 11 genera of Triticum. The evolutionary relationships herein identified were consistent with those of previous studies based on nuclear DNA markers and, to some extent, cp genome data [11,13,14,24,51,52,53,63,64]. According to Sha’s research, *Psathyrostachys* is the donor of the Ns genome in *Leymus* species [14]. What needs more attention is that the phylogenetic tree based on cp genome sequences existed with minor differences from the phylogenetic tree based on shared protein-coding genes. We inferred that the reason for this is barcode genes laid in the cp genome, and the result is consistent with Liu’s and Guo’s research [11,16]. What is more, the close relationship between *Elymus* and *Leymus* may also be related to their origin of genome [11]. Here, we provide further evidence based on plastome data and protein-coding genes. The results of our phylogenetic analysis indicate that the cp genome can be used as a useful tool to explore species relationships.

## 5. Conclusions

Based on 13 newly sequenced and three previously reported *Leymus* cp genomes, we have found that the plastome is highly conserved and similar concerning its overall structure within this genus. Our findings demonstrate that most SSRs are A/T rich and are more commonly located in non-coding regions. The cp genome sequence identity is highly similar among *Leymus* species, especially in the protein-coding and IR regions. The differences identified in the CDS and IGS regions of *Leymus* species revealed 10 highly variable regions that may be used as new genetic markers for DNA barcoding and phylogeny research. In addition, comparisons of the IR boundaries further showed that the IR contraction and expansion events in *Leymus* species lead to different genomic locations of the *rpl22*, *rps19*, *ndhH,* and *psbA* genes. The close relationship between *Leymus* and *Psathyrostachys* highlighted by a phylogenetic tree design based on plastome data further indicated that as early as in the 1980s, *Psathyrostachys* may be the donor of the Ns genome to *Leymus* species. Taken together, these findings provide useful information for the identification and phylogenetic analysis of *Leymus* species.

## Figures and Tables

**Table 1:** Number of SSRs in the chloroplast genomes of 16 *Leymus* species.

Species	Total SSRs	Compound SSRs	Types					LSC	SSC	IRa	IRb
			Mono-	Di-	Tri-	Tetra-	Penta-				
*Leymus angustus*	39	2	26	3	3	6	1	33	4	1	1
*Leymus arenarius*	47	3	29	6	3	9		37	4	3	3
*Leymus chinensis*	48	3	30	6	3	9		36	4	4	4
*Leymus cinereus*	43	2	22	6	3	9	3	33	2	4	4
*Leymus condensatus*	41	2	28	4	2	6	1	36	3	1	1
*Leymus coreanus*	50	5	30	6	3	9	2	40	2	4	4
*Leymus duthiei*	36	2	19	6	2	9		27	3	3	3
*Leymus karelinii*	49	3	31	6	3	9		36	3	5	5
*Leymus komarovii*	43	6	23	6	3	10	1	33	2	4	4
*Leymus mollis*	37	1	24	4	2	6	1	31	4	1	1
*Leymus multicaulis*	47	3	29	6	3	9		36	3	4	4
*Leymus paboanus*	44	1	26	6	3	9		31	5	4	4
*Leymus racemosus*	49	3	31	6	3	9		37	4	4	4
*Leymus ramosus*	47	2	29	6	3	9		35	4	4	4
*Leymus secalinus*	46	2	28	6	3	9		34	4	4	4
*Leymus triticoides*	41	2	20	6	3	9	3	31	2	4	4
Total	707	42	425	89	45	136	12	546	53	54	54

## Data Availability

The complete chloroplast genome sequences of the 13 species we sequenced were deposited at NCBI, GenBank accession number: MZ595315, NC_044900.1, MZ595314, MZ595313, MZ595312, MZ595316, MZ595311, MZ595317, MZ595318, MZ595319, MZ595320, MZ595321 and MZ595310. Row data are available at SRA under the accession number: SRR19118231, SRR13308283, SRR19118230, SRR19118227, SRR19118226, SRR19118225, SRR19118224, SRR19118223, SRR19118222, SRR19118221, SRR19118220, SRR19118229 and SRR19118228.

## Supplementary Materials

The following are available online at https://www.mdpi.com/article/10.3390/genes13081425/s1, Table S1: The information of *Leymus* species in this study; Table S2: Gene composition in *Leymus* chloroplast genome; Table S3: Summary of chloroplast genome characteristics in *Leymus* species; Table S4: Numbers of identified SSRs motifs in the 16 *Leymus* chloroplast genomes; Table S5: SSRs location in the 16 *Leymus* chloroplast genomes; Table S6: Eta, Pi value, and PICs of Protein-coding regions(CDS) in *Leymus* species; Table S7: Eta, Pi value, and PICs of non-coding regions in *Leymus* species.
